# Genome streamlining to improve performance of a fast-growing cyanobacterium *Synechococcus elongatus* UTEX 2973

**DOI:** 10.1128/mbio.03530-23

**Published:** 2024-02-15

**Authors:** Annesha Sengupta, Anindita Bandyopadhyay, Debolina Sarkar, John I. Hendry, Max G. Schubert, Deng Liu, George M. Church, Costas D. Maranas, Himadri B. Pakrasi

**Affiliations:** 1 Department of Biology, Washington University, St. Louis, Missouri, USA; 2 Department of Chemical Engineering, Pennsylvania State University, State College, Pennsylvania, USA; 3 Wyss Institute for Biologically Inspired Engineering, Harvard University, Cambridge, Massachusetts, USA; 4 Department of Genetics, Harvard Medical School, Boston, Massachusetts, USA; University of Washington School of Medicine, Washington DC, USA

**Keywords:** CRISPR-Cas3, genome minimization, cyanobacteria, large progressive deletion, essential gene identification

## Abstract

Cyanobacteria are photosynthetic organisms that have garnered significant recognition as potential hosts for sustainable bioproduction. However, their complex regulatory networks pose significant challenges to major metabolic engineering efforts, thereby limiting their feasibility as production hosts. Genome streamlining has been demonstrated to be a successful approach for improving productivity and fitness in heterotrophs but is yet to be explored to its full potential in phototrophs. Here, we present the systematic reduction of the genome of the cyanobacterium exhibiting the fastest exponential growth, *Synechococcus elongatus* UTEX 2973. This work, the first of its kind in a photoautotroph, involved an iterative process using state-of-the-art genome-editing technology guided by experimental analysis and computational tools. CRISPR-Cas3 enabled large, progressive deletions of predicted dispensable regions and aided in the identification of essential genes. The large deletions were combined to obtain a strain with 55-kb genome reduction. The strains with streamlined genome showed improvement in growth (up to 23%) and productivity (by 22.7%) as compared to the wild type (WT). This streamlining strategy not only has the potential to develop cyanobacterial strains with improved growth and productivity traits but can also facilitate a better understanding of their genome-to-phenome relationships.

**IMPORTANCE:**

Genome streamlining is an evolutionary strategy used by natural living systems to dispense unnecessary genes from their genome as a mechanism to adapt and evolve. While this strategy has been successfully borrowed to develop synthetic heterotrophic microbial systems with desired phenotype, it has not been extensively explored in photoautotrophs. Genome streamlining strategy incorporates both computational predictions to identify the dispensable regions and experimental validation using genome-editing tool, and in this study, we have employed a modified strategy with the goal to minimize the genome size to an extent that allows optimal cellular fitness under specified conditions. Our strategy has explored a novel genome-editing tool in photoautotrophs, which, unlike other existing tools, enables large, spontaneous optimal deletions from the genome. Our findings demonstrate the effectiveness of this modified strategy in obtaining strains with streamlined genome, exhibiting improved fitness and productivity.

## INTRODUCTION

Cyanobacteria are the most ancient and abundant oxygenic photosynthetic organisms that are largely responsible for the Earth’s viable environment (1). These photosynthetic prokaryotes have been identified as potential platforms for sustainable carbon-neutral bioproduction because of their unique ability to harvest sunlight as their sole energy source for converting greenhouse gases (carbon dioxide) into value-added chemicals. This bioprocess in theory is also economically sustainable as it enables free and ubiquitous substrates to enter the bio-economy. In comparison to other photoautotrophs, cyanobacteria have several advantages, and, therefore, efforts are ongoing to understand and develop the cyanobacterial platform as sustainable bio-factories (2–5). However, relatively slower growth and limited knowledge of their genomic traits as compared to heterotrophs such as *Escherichia coli* or yeast have restricted progress in this direction. Although the recent isolation of a few fast-growing strains has made cyanobacterial-based bioproduction more compelling and tractable than ever, concerted efforts are needed to get their productivity at par with their heterotrophic counterparts (6–10). Although most commonly studied cyanobacterial genomes are generally smaller in size than that of *E. coli*, they exhibit cryptic metabolic and regulatory features, owed likely to their photosynthetic lifestyle, unique evolutionary history, and adaptation to various unfavorable environmental conditions (11).

The main aim of this study was to test the feasibility of employing the genome reduction strategy as a means to shed excess biological complexity and simplify the genome of a fast-growing cyanobacterium without compromising its desirable traits. The clade *Synechococcus elongatus* hosts all of the fast-growing cyanobacterial strains identified to date (8), and a genome minimization approach will be beneficial for unraveling the genome-level function and the overall metabolism of these strains, which in turn will aid their development into cell factories. The goal is not to obtain a truly minimum genome for a photosynthetic organism but rather to identify and remove genes dispensable under bioproduction-relevant conditions (high light and CO_2_) without compromising growth and productivity.

Genome streamlining is a natural evolutionary process of eliminating non-beneficial genes, since a smaller genome reduces the metabolic burden on the cell and improves fitness (12–14). As an engineering strategy, it has been successfully employed in model heterotrophs leading to improved fitness and performance (15–18). A genome reduction of 25% in *E. coli* led to a 1.6-fold improvement in growth rate and improved recombinant protein production (19, 20). Similarly, genome streamlining in a *Pseudomonas* strain resulted in several appealing traits such as faster growth, increased biomass production, enhanced plasmid stability, and, overall, a more efficient energy metabolism (21, 22). These studies indicate that a chassis strain with a streamlined genome avoids the unnecessary burden of replicating and expressing genetic elements that are not useful under production conditions. Reducing this unnecessary genetic burden may ensure that more cellular resources are available for the expression of heterologous pathways. By removing genes of unknown function, it also creates a chassis organism that is more fully understood and more amenable toward genetic engineering and synthetic biology efforts (16). Despite the favorable outcomes of genome minimization in heterotrophs (20–23), limited efforts have been made to implement such strategies in phototrophs. So far, two reports explore genome streamlining. Removing ~2% (118 kb) of the genome of *Anabaena* PCC 7120 has been demonstrated using the CRISPR-Cas12a system for targeted deletions, but this study did not explore the effects of these edits on strain performance (24). Deletion of several large fragments of DNA from the genome of *S. elongatus* PCC 7942 has been performed, producing a septuple mutant with approximately 3.8% of its genome removed. These mutants were further studied to understand the changes in the transcriptomic profile of the cells resulting from the deletions. The CRISPR-Cas12a-editing tool was used to obtain the targeted, specified, and markerless deletions in this study (25). Although Cas12a is a dynamic and versatile tool, it does not allow the flexibility to explore and identify unknown stretches of dispensable and indispensable regions in the genome of a strain. Recently, a novel Class I multi-Cas protein was commissioned for large deletions in heterotrophs. The dual helicase and exonuclease activity of Cas3 enabled large simultaneous bidirectional deletions, without the necessity of a repair template (26). Recently, in one of our studies, we have successfully commissioned an inducible CRISPR-Cas3 system in *S. elongatus* UTEX 2973 to truncate the light-harvesting antenna structure for maximizing fitness and productivity under specified condition (27). However, this tool has not been explored for genome streamlining in cyanobacteria. Therefore, it is of interest to investigate this Cas system and exploit its beneficial features for genome minimization of photoautotrophs.

**Fig 1:**
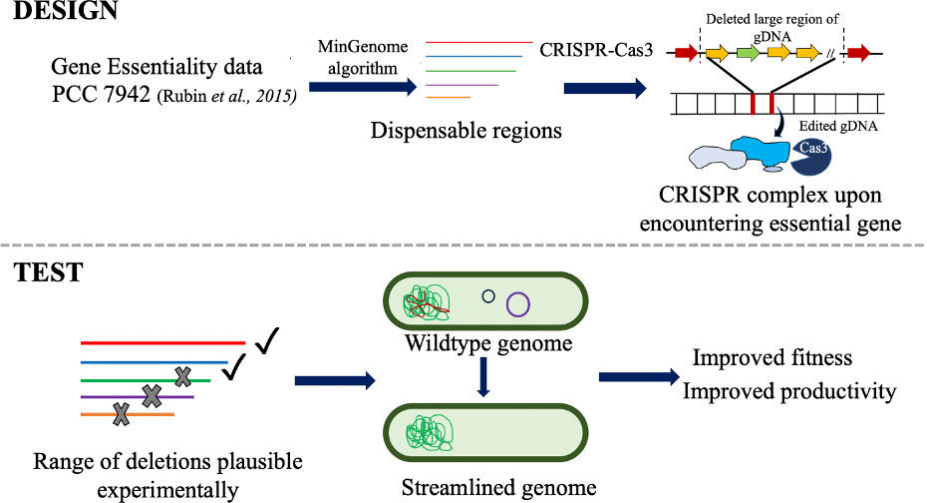
Schematics showing the strategy used for systematic genome streamlining in cyanobacteria. A combinatorial approach integrating computational (design) and experimental (test) tools to first identify the dispensable regions in *S. elongatus* UTEX 2973 and further use novel CRISPR tool to validate the prediction and create a strain with improved fitness and productivity.

In this study, we investigate the effect of systematic reduction of dispensable regions from the genome of *Synechococcus* 2973, the fastest growing, high light thriving cyanobacterium that exhibits high sucrose production titer (6, 10). We first identified five large stretches of dispensable genomic regions in this strain using the MinGenome algorithm (28) and then commissioned the novel CRISPR-exonuclease system, CRISPR-Cas3 to achieve flexible progressive large deletions. CRISPR-Cas3, besides deleting large regions, enabled identification of non-dispensable genes in *Synechococcus* 2973, which otherwise were predicted as dispensable. We successfully deleted the optimal stretch of two of the five dispensable regions identified by our *in silico* analysis. The two large deletions were combined to obtain a strain with genome reduction of 55 kb. The strains with a reduced genome showed improved growth and sucrose productivity. This proof-of-concept study (Fig. 1) demonstrates that systematic minimization of cyanobacterial genomes has the potential to develop these organisms as super-strains that might hold the potential to boost a carbon-neutral bio-economy and mitigates climate issues.

## RESULT

### *In silico* prediction of probable dispensable regions from the genome of *Synechococcus* 2973

The first step toward streamlining the genome of *Synechococcus* 2973 was to identify the dispensable genomic regions (Fig. 1). Since *Synechococcus* 2973 shares >99% genomic identity with the model strain *Synechococcus* 7942, the list of essential genes already available for *Synechococcus* 7942 (29) was borrowed and mapped to *Synechococcus* 2973 using a bidirectional protein blast with a stringent *e*-value cut-off of 10^−10^ to avoid spurious hits. The MinGenome algorithm (28) was employed to predict large dispensable regions with three criteria: (i) The growth rate of the strain is fixed at the maximum, (ii) essential genes mapped from *Synechococcus* 7942 cannot be deleted, and (iii) find the longest stretch first and iterate to find the consecutive ones. This strategy predicts five large regions of the genome that can be dispensed without affecting the strain fitness (Table 1). The list of gene annotations of each cluster is provided in Tables S1 to S5.

**TABLE 1:** A list of large gene clusters that are predicted to be dispensable for growth of *Synechococcus* 2973^*a*^

Rank	Start	End	Length (bp)
1	M744_12940	M744_13140	33,952
2	M744_10800	M744_10895	26,399
3	M744_02780	M744_02900	25,699
4	M744_12500	M744_12615	22,544
5	M744_05410	M744_05555	22,235

^*a*^ The predicted dispensable region in between the start site of the first gene of the cluster and the start site of the end gene of the cluster.

### CRISPR-Cas3-mediated genome editing enabled the deletion of large genomic regions

**Fig 2:**
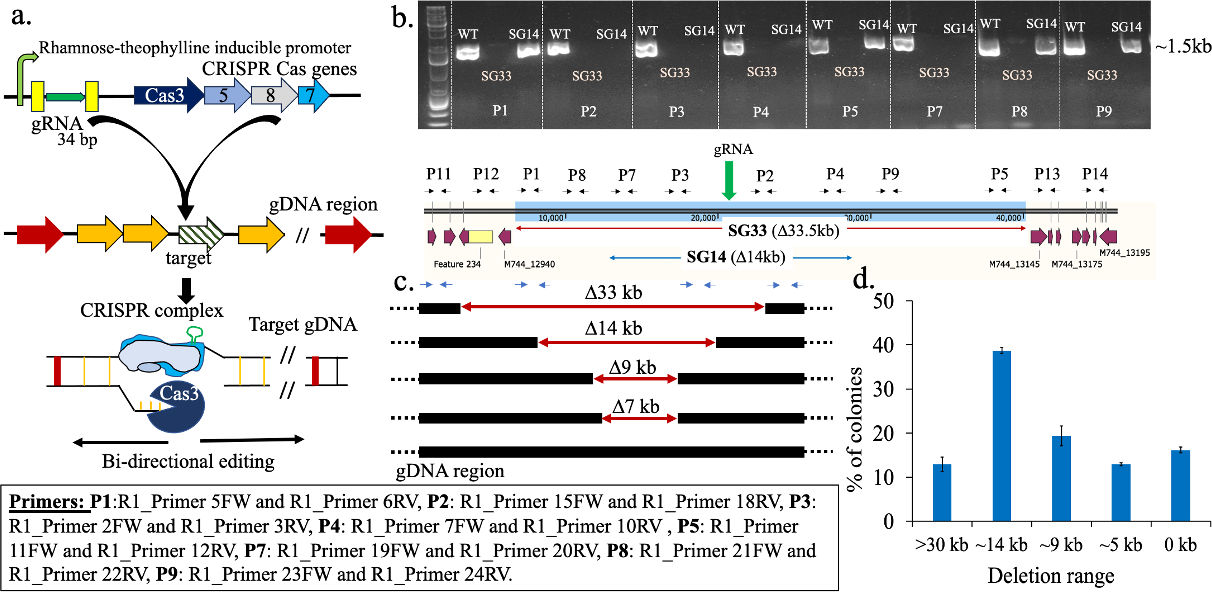
Development of the CRISPR-Cas3 system for spontaneous large deletions in cyanobacteria. (**a**) Schematics showing the functioning of the CRISPR-Cas3 system. (**b**) The result from tiling PCR (gel picture) using different primer sets (P1–P9) showing the absence or presence of a 1.5-kb band for two colonies (SG33 and SG14). The absence of a band indicates that the region is deleted from the strain as compared to WT. (**c**) The length of deletions obtained when the CRISPR system was targeted at the R1 region. (**d**) Frequency of occurrence of colonies of a particular length of deletion. The error bars represent the variation observed between the differences in the number of colonies obtained for a particular length of deletions between three separate experiments.

A rhamnose-theophylline-inducible Class I CRISPR system involving multi-Cas proteins was employed to streamline the genome of *Synechococcus* 2973 (Fig. 2a). In this CRISPR system, Cas3 is the key component exhibiting both helicase and nuclease activity, enabling large bidirectional deletions. The other Cas proteins (Cas5, 7, and 8) help in maintaining the fork structure required for these long deletions (26, 27). Unlike Cas12a-mediated editing where the length of deletion is predetermined based on existing knowledge of the region of interest (designed repair template), the Cas3 system allows spontaneous deletion of DNA stretches on either side of the targeted region, as long as the deletions are not detrimental for the strain. Therefore, the Cas3 CRISPR system has the potential to not only identify previously unknown dispensable regions in the genome but also uncover essential genes within a stretch of DNA that has been computationally determined as dispensable, thereby averting any adverse effects on the strain from large-scale deletion experiments.

In our previous study, as a proof of concept, the inducible CRISPR-Cas3 system was tested to delete dispensable genes from the genomic cluster encoding the light-harvesting antenna complex of *Synechococcus* 2973. Interestingly, every deletion attempt led to a strain with a reduction of 4 kb. Investigation of the region beyond 4 kb revealed that the genes immediately flanking the deletion on either side were designated as essential as per the gene essentiality data (27). These experiments revealed the potential of the Cas3 tool in identifying essential genes in a stretch of DNA while simultaneously identifying dispensable regions. Thus, our next goal was to test the feasibility of using the Cas3 entourage for deletions of large genomic segments from cyanobacterial genomes, analogous to what has been achieved in heterotrophs. In this work, a strategy similar to a heterotrophic study (27) was implemented to target all the five regions predicted as dispensable by our *in silico* analysis (Table 1). We first commissioned CRISPR-Cas3 for the largest stretch identified, R1 (Table 1), in order to optimize the system for large deletion in *Synechococcus* 2973 (Fig. 2). A 34-bp-long guide RNA (gRNA) was designed for R1 that targeted the gene M744_13025 (which is approximately at the midpoint of the predicted region). Around 30 colonies out of 1,000 were initially screened for deletions by using tiling PCR with three sets of primers (P1, P3, and P4), and around 20% of the colonies showed no deletions (Fig. S1). The probable position of the primers initially chosen is shown in Fig. 2b. A more extensive tiling PCR with multiple primers helped identify the range of deletions in different colonies, such as SG33 and SG14 (Fig. 2b). Similar analysis of all the rest 80% positive colonies indicated a wide range of deletions from 0 to >30 kb (Fig. 2c). A frequency analysis showed that around 40% of the deletions were in the range of 14–15 kb, and ~10% colonies showed >30-kb deletion (Fig. 2d). This demonstrated that the CRISPR-Cas3 tool has the potential to execute a large range of deletions in cyanobacteria, generating a library of random deletions.

**Fig 3:**
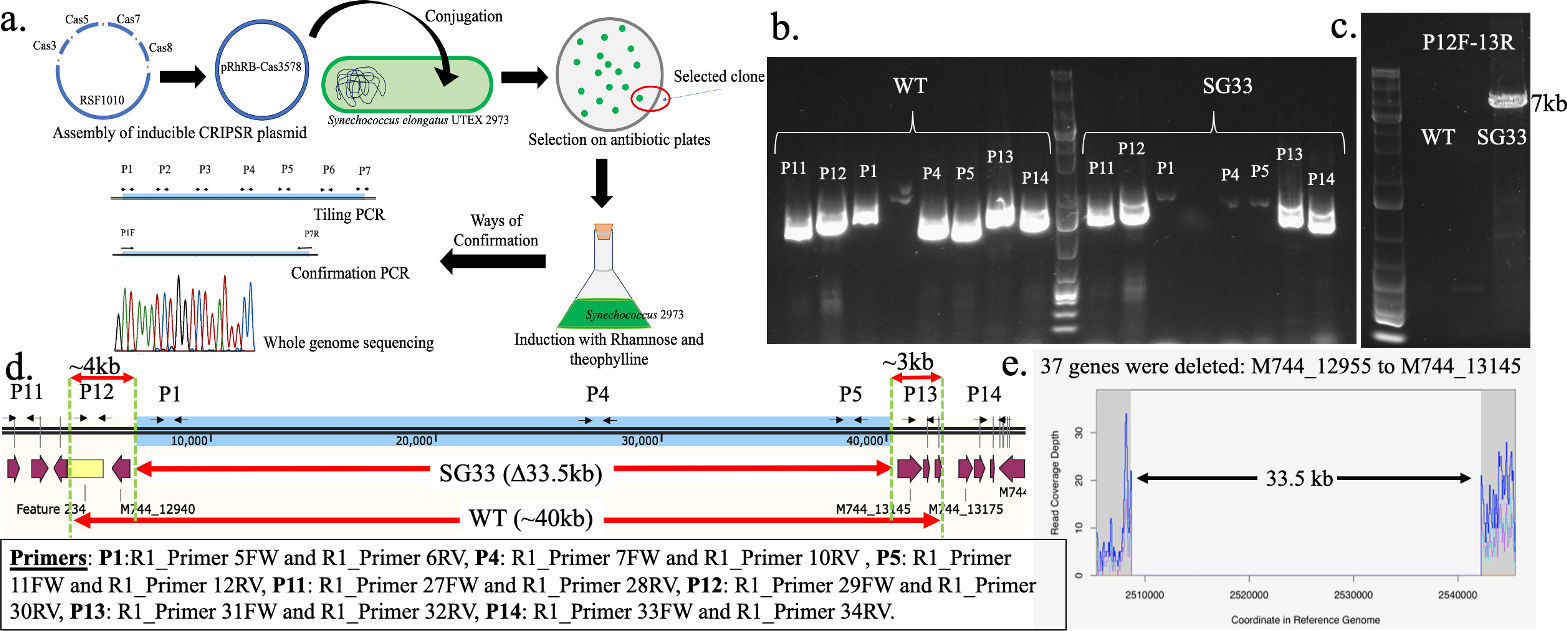
Generation of the SG33 strain using CRISPR-Cas3 system. (**a**) The workflow for obtaining the markerless strain with the minimized genome. (**b**) The gel picture showing the absence or presence of 15-kb band when amplified with different primer sets in a tiling PCR to identify the deleted regions. (**c**) PCR confirming the presence of a 7-kb band in the SG33 strain instead of the 40-kb band expected in the WT. The large 40 kb band could not be obtained in WT. (**d**) Schematics showing the R1 region and the range of deletions and the regions of primer binding. (**E**) The whole-genome sequencing of the SG33 strain showed a clean and segregated deletion.

This tested inducible CRISPR-Cas3 system was then used to create strains with streamlined genome by targeting the predicted regions (Table 1). Figure 3a briefly shows the workflow for generating these deletion mutants using this inducible CRISPR-Cas3 system. CRISPR-mediated targeting of the R1 region gave rise to a markerless strain 2973∆33.5 kb showing a deletion of 33.5-kb region (hereafter mentioned as SG33) (Fig. 3). The extent of deletion in the strain was initially confirmed by tiling PCR (Fig. 3b), and then, the total length of deletion was determined by confirmation PCR using set of primers that were previously determined based on the tiling PCR results (Fig. 3b). Using primers flanking the 40-kb targeted region, a band of ~7 kb was obtained in the SG33 strain, indicating a large deletion of ~33 kb (Fig. 3c and d). Finally, whole-genome sequencing confirmed the range of deletion to be 33.5 kb (Fig. 3e). The whole-genome sequencing also confirmed no additional mutations in the SG33 strain.

**Fig 4:**
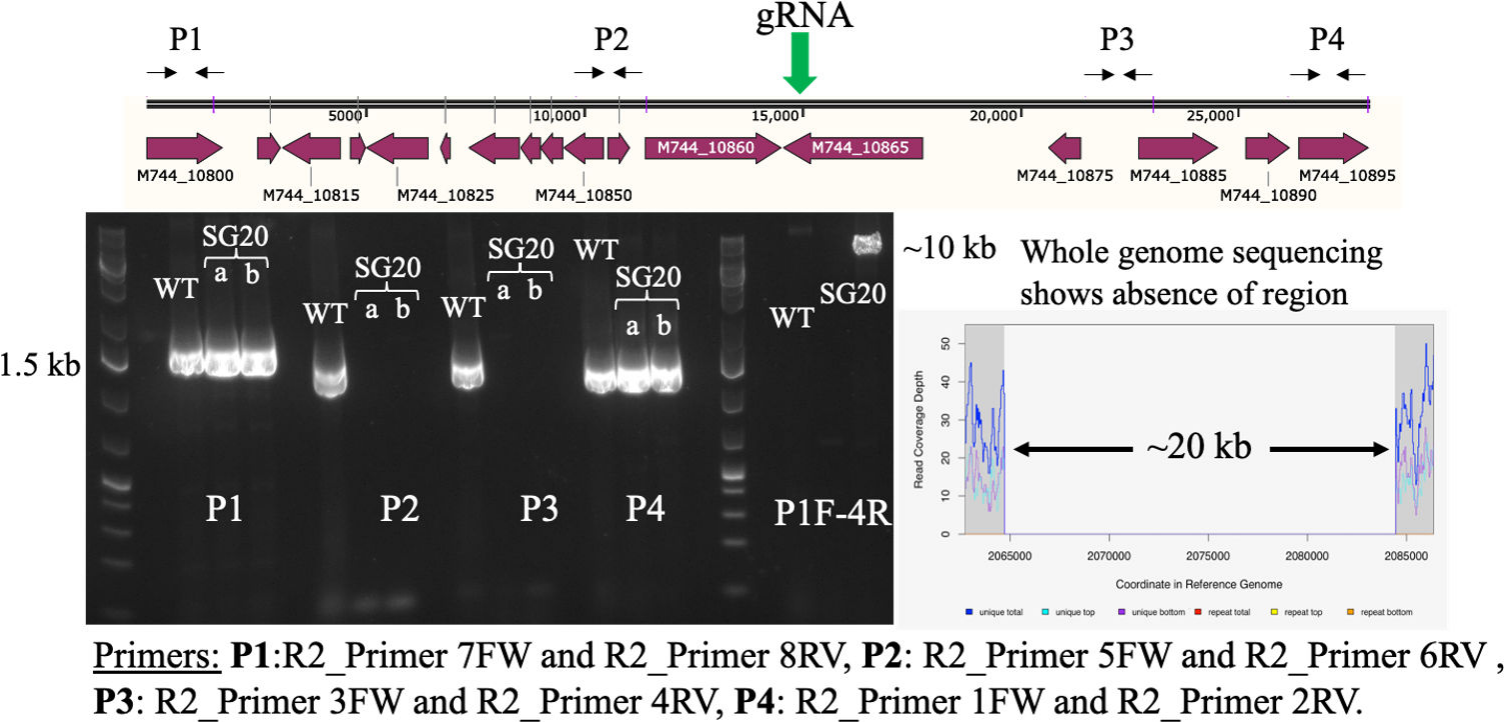
Generation of the SG20 strain. The schematic showing the R2 region and the primer binding sites. The gel picture is of the tiling PCR performed using the different primer sets (P1–P4) and the confirmation gel using the primers P1 forward and P4 reverse. A 10-kb band is visible instead of the 30-kb band in SG20 as compared to the WT. No band is visible as 30 kb is difficult to amplify and visualize. A snap of the whole-genome sequencing chromatogram shows the absence of reads around the R2 region. Two different colonies with deletion were tested (**a, b**) as shown in the gel picture.

Similar workflow was applied for the R2 region, which led to its partial deletion. Although this region was predicted to be ~26.4 kb long (Table 1), experimentally, the largest deletion obtained was 19.7 kb, giving rise to the strain 2973∆19.7 kb (hereafter mentioned as SG20) (Fig. 4). While the first two regions, R1 and R2, could be deleted (Fig. S2), the other identified regions (R3, R4, and R5) could not, despite several attempts with different gRNA and protospacer adjacent motif (PAM) sequences (Fig. S3). We then attempted to delete these regions using the CRISPR-Cas12a system with two gRNAs (24, 30) but remained unsuccessful as well (data not shown). This suggested that the regions predicted as dispensable based on S7942 essentiality data are probably not accurate enough and indicated the need for *Synechococcus* 2973-specific essentiality data.

We then proceeded to combine the two large deletions (SG33 and SG20) to obtain a single strain with a large reduction in the genome. Initially, SG33, the strain with the largest deletion, was used as the base strain to tier the next largest deletion. However, the conjugation efficiency of this strain was very poor [1,000-fold lower than the WT or the SG20 strain (data not shown)]. Therefore, we decided to use the SG20 strain as the base strain into which the plasmid carrying the Cas3 machinery for R1 deletion was introduced. Whole-genome sequencing of one of the transformant colonies showed a total deletion of 55 kb in this strain (henceforth referred to as SG55) (2973∆19.7 kb∆34.3 kb). Unlike SG33, where 33.5 kb was deleted upon targeting R1, a 34.3-kb region could be deleted when the same region was targeted in the SG20 strain. The above results confirmed the efficacy of the CRISPR-Cas3 genome-editing tool in creating spontaneous and large deletions in cyanobacteria.

### Minimization of genome showed improved growth and productivity

**Fig 5:**
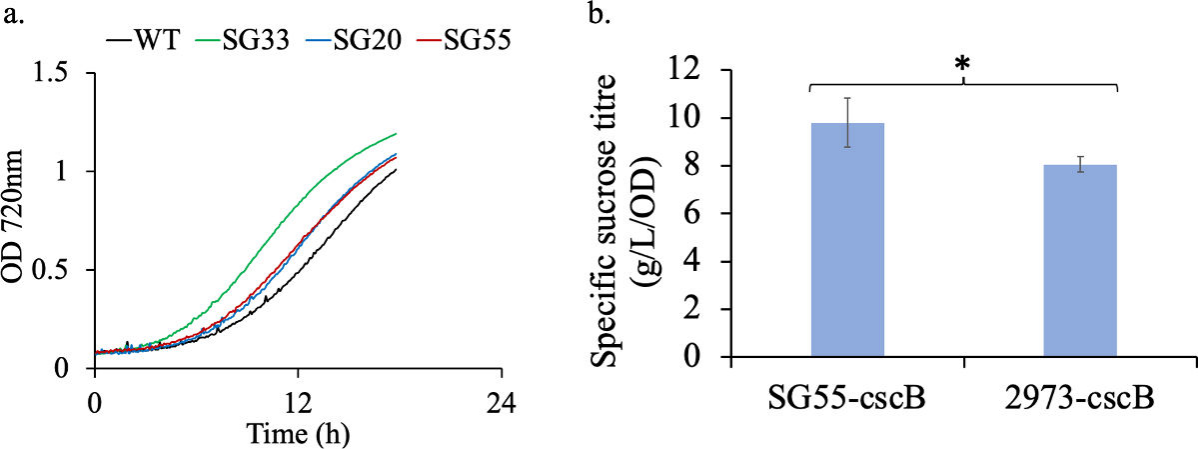
Characterization of the engineered strains with minimized genome. (**a**) Growth comparison of WT, SG33, SG20, and SG55 under high light and high CO_2_. Experiments were performed in triplicates (*n* = 3). Representative growth curves are shown. (**b**) Sucrose productivity of SG55 as compared to WT. Experiments were performed in triplicates (*n* = 3), and the error bars correspond to standard deviations from at least three biological replicates. Asterisk (*) denotes statistically different values of μ (*P* < 0.05).

The strains with streamlined genome were characterized to determine the fitness and photosynthetic productivity. Since our primary goal was to develop a reduced genome cyanobacterial host that can thrive under high light and high CO_2_, all studies were performed under high light (1,500 µmol·m^−2^·s^−1^) and high CO_2_ (1% CO_2_ bubbling) conditions. A comparative analysis of growth rates revealed an increase of 23%, in the SG33 strain compared to the WT. In contrast, a growth rate increase of only 5% was observed in SG20, while SG55 showed an improvement of 9% (Fig. 5a; Table S6). However, this enhancement in growth rate was not evident under conditions where carbon was a limiting factor (Fig. S4). Interestingly, the SG55 strain showed a 10-fold higher transformation efficiency with RSF1010 plasmids compared to the WT. Analysis of the genome sequence of this strain indicated that this increase in efficiency could likely be due to a mutation it acquired in SeAgo, an argonaute protein known to reduce the efficiency of RSF1010 plasmid transformation (31).

In addition to the large chromosomal deletions, the two endogenous plasmids pANL (~50 kb) and pANS (~7 kb) were also deleted from the WT strain. Since these deletions did not show any added growth advantages (Fig. S5), these deletions were not tiered into the SG55 strain. Analysis of the photosynthetic efficiency of the strains with streamlined genomes revealed no difference in quantum yield (Fv/Fm 0.35 ± 0.08) as compared to the WT, indicating no adverse impact of the deletions on key physiological processes. Thus, the streamlining strategy employed in this study reduced the metabolic burden in the WT *Synechococcus* 2973 strain resulting in faster growth but did not disrupt its basic cellular functions.

The final strain with streamlined genome (∆55 kb) and improved phenotypic characteristics was engineered to secrete sucrose by integrating *cscB*, a heterologous sucrose transporter gene (10, 32) into the genome. The engineered SG55 strain (SG55-cscB) showed 22.7% higher sucrose production as compared to the strain expressing the transporter alone (10) (Fig. 5b). Our findings demonstrate that genome streamlining in cyanobacteria can be a promising strategy for improving both productivity and fitness, provided the correct genes are dispensed.

### Analysis of the deleted regions revealed a large number of hypothetical genes

Streamlining of genome is an effective strategy for developing a strain as a production host with beneficial features, but analysis of the dispensable genes might also help deduce the role of certain genetic elements. In our analysis, we observed that in the predicted dispensable regions, >50% of genes are annotated as hypothetical and majority of these hypothetical genes are in R1 and R2 (Tables S1 to S5); therefore, deciphering the underlying reason for the observed phenotype of the deleted strain was not possible with certainty.

Since, in this study, the gene essentiality data were borrowed from *Synechococcus* 7942 (29), there are some discrepancies between the experimentally obtained deletions and predicted dispensability data. To exemplify, in the R1 region, there are 37 genes (M744_12940–M744_13135), and the genes flanking this region have been categorized as essential. Conversely, our sequencing results for the SG33 strain that has the R1 deletion showed the deletion of M744_13140, a gene annotated as essential. Although, for non-model strains, strain-specific essentiality data are important, the use of the novel Cas3 system provided the flexibility to dispense the optimal length of regions from the genome for improved fitness. Therefore, the genome streamlining with the CRISPR-Cas3 system is an advantageous strategy, and a more high-throughput streamlining effort might help create a library of strains exhibiting varied phenotypes.

## DISCUSSION

Genome streamlining is a synthetic biology approach that allows strategic reduction of the genome for attaining a desirable strain phenotype, and this approach has been demonstrated to be successful in heterotrophs. There are two approaches of genome streamlining, bottom-up and top-down (33). Although most minimized genome studies have employed a bottom-up strategy to create a truly minimal genome from scratch (16, 34), this strategy can pose several challenges as it demands a vivid and thorough knowledgebase of all biological processes and interactions and requires efficient synthetic DNA synthesis and assembly tools. Moreover, cyanobacteria being a polyploid organism exponentially enhance the challenge of introducing and maintaining the synthetic chromosome. The other approach is top-down, which involves systemic streamlining of the existing genome based on prior information regarding the core essential genes. Most genome streamlining efforts have relied on traditional and, more recently, the CRISPR-Cas12a/9-mediated genome-editing tools (20, 21, 24, 25). For strains where the essentiality information is available, the strategy to delete fixed, specified regions of the genome is advantageous as it leaves less room for casualties such as drastic loss of fitness. However, even with prior knowledge, the experimental outcome might not correlate with *in silico* predictions. Like, in *E. coli* MG1655, a reduction of 29.7% exhibited severely impaired phenotype, while a 7% reduction showed no retarded phenotype (20). Therefore, for known and, more importantly, for newly discovered strains, the spontaneity or randomness in the extent of deletion might be the key to obtain a strain with enhanced features, and CRISPR-Cas3-mediated genome editing has the potential (26, 27). The RNA-guided Cas3 protein has dual helicase-nuclease activity that allows large, progressive, random deletion of genomic region unless encountered with an essential gene. CRISPR-Cas3 has been demonstrated to be effective in heterotrophs, such as *Pseudomonas aeruginosa*, where deletion of genomic regions as large as 424 kb with a mean of 92.9 kb and median of 58.2 kb deletion (26) was observed. Therefore, its use in photoautotrophs for streamlining and other large-scale editing purposes is worth exploring.

In this study, we focused on developing a genome streamlining strategy for a non-model cyanobacterium *Synechococcus* 2973 to obtain a strain with reduced genome and improved fitness. *Synechococcus* 2973 is the fastest-growing cyanobacterium known so far with a doubling time comparable to heterotroph model strains such as yeast (6, 35). This organism has the potential to be developed as the next-generation production host; however, the complexity of the strain poses challenges for major engineering efforts. We attempted to minimize the metabolic burden on this strain by first identifying dispensable genomic regions using the MinGenome algorithm (28) and then removing them under bioproduction-relevant conditions without compromising growth and productivity (Fig. 1). This iterative integrated approach led to the creation of engineered *Synechococcus* 2973 strains with minimized genomes exhibiting significant growth advantage (Fig. 5a). Our results indicate that the extent of growth advantage is not dependent on the extent of genome reduction but on the set of deleted genes. Our analysis revealed that some genes in R1 are predicted as phage-associated proteins. Although the SG55 strain has the largest range of deletion (55 kb), the growth improvement is more in SG33 (33.5-kb deletion), and this might be due to the deletion of prophage-like genes. A previous study in *Vibrio natriegens* revealed that the deletion of prophage-containing genomic regions is an effective engineering strategy for improving growth (36).

Since a majority of the genes are annotated as hypothetical, further analysis to decipher the genetic features responsible for the phenotype was not possible. We tested the effect of minimization on productivity by engineering SG55 strain for sucrose overproduction and observed a 22.7% improvement in sucrose titer (Fig. 5b) as compared to sucrose-producing WT (10). This strategy of streamlining the genome for improved growth and phenotype might come at the cost of robustness under non-controlled conditions such as outdoor-like conditions (carbon limited). Under the carbon-limited condition, the engineered strains failed to show the improved phenotype (Fig. S4). This decreased fitness in conditions mimicking the natural environment (limited carbon available) might be a boon as it lessens the risk of their release and the chance to overtake the WT populations. However, in under elevated CO_2_, these strains might be advantageous to utilize more available carbon.

A more high-throughput understanding of the phenotype and genotype relationship would provide a better insight into the complexity of the strains. The stochastic nature of the CRISPR-Cas3 system offers the potential to discern the essentiality of genes in the targeted regions and obtain an optimal dispensable stretch while retaining or enhancing strain fitness. This hybrid strategy of combining computational analysis with progressive deletion of genomic regions can generate a library of engineered model and non-model photoautotrophic strains with streamlined genomes, thereby paving the path for developing these remarkable green hosts into predictable bio-systems with the potential to mitigate climate problems and boost bio-economy.

## MATERIALS AND METHODS

### Computationally predicting the longest dispensable regions

To identify regions in the *Synechococcus* 2973 genome that can be deleted without affecting organism growth, we used a genome reduction algorithm called MinGenome (28). MinGenome uses a mixed-integer linear programming-based approach to systematically identify (in descending order) all contiguous dispensable regions. First, essential genes were identified using sequence homology with *Synechococcus* 7942 using a bidirectional protein BLAST. These can then be flagged and their deletion prohibited when implementing MinGenome. Next, we used flux balance analysis on the published genome-scale metabolic (GSM) model for *Synechococcus* 2973 (37) to compute the maximum growth rate using 10 mmol/gDW h CO_2_ (as a basis) and default model parameters. MinGenome was then implemented using this metabolic model while fixing the growth rate at the determined maximal value and constraining all essential genes to be preserved. The longest contiguous genome regions to be deleted were thus iteratively identified. It should be noted that as long as the GSM is used to simulate carbon-limited phototrophic growth, the deletion regions identified here should be invariant of the specific model parameters used.

### Chemicals and reagents

All enzymes were purchased from New England Biolabs (NEB, Ipswich, MA, USA) and Thermo Fisher Scientific (Waltham, MA, USA). The molecular biology kits were obtained from Sigma-Aldrich (St. Louis, MO, USA). The chemicals, reagents, and antibiotics used in this study were of analytical/High-performance liquid chromatography (HPLC) grade and were procured from Sigma-Aldrich (St. Louis, MO, USA). The primers were ordered from Integrated DNA Technologies (IDT, Coralville, IA, USA). The plasmids were sequenced by Genewiz (South Plainfield, NJ, USA).

### Cultivation condition of the WT and mutants

The WT *Synechococcus* 2973 strain and mutant strains were cultivated and maintained in the Caron chamber under 300 µmol·m^−2^·s^−1^ of light and 0.5% CO_2_ at 38°C and 250 rpm. *E. coli* containing specific plasmids was cultivated overnight at 37°C in Luria-Bertani Broth (LB) supplemented with appropriate antibiotic. Freezer stocks were maintained at −80°C in 7% DMSO for cyanobacterial strains and 25% glycerol for *E. coli* strains.

### Development of Cas3-mediated large random deletions

The engineered strains were constructed using the CRISPR-Cas3 editing technique novel to the cyanobacterial system. This is a multi-Cas system where Cas3 has the nuclease-helicase activity (26). In our previous study, we developed an inducible CRISPR plasmid pSL3577 (pRhRB-Cas3578), where the *cas* genes and the gRNA are controlled using the rhamnose inducible promoter and theophylline inducible riboswitch (Fig. S6) (27, 38). The BsaI restriction site was used as the site for gRNA cloning in pSL3577, and this system does not require any predetermined repair template. The recombinant plasmid (pSL3578) was conjugated into the *Synechococcus* 2973 by triparental mating using a modified protocol. Briefly, the recombinant plasmids were transformed into the competent cells of WM6026 containing the pRL623 plasmid. This strain was used for conjugation. Two milliliters of *E. coli* cells were grown overnight in SOB media supplemented with 30-µg/mL chloramphenicol and 250-mM DAP, which was diluted in 25-mL SOB and DAP (without antibiotic) and grown to an OD of 0.1–0.2. This 25 mL of exponentially grown conjugal strain was mixed with 5 mL of exponentially grown cyanobacterial culture and centrifuged at 2,000 rpm. The mixed cells were washed gently and resuspended in 400 µL of BG-11 medium and evenly spread onto a HATF nitrocellulose filter paper (MilliporeSigma, St. Louis, MO, USA) that was placed on a BG-11 5% LB agar plate without antibiotic, prior to the experiment. The plates were incubated for 24 h under 100 µmol·m^−2^·s^−1^ of light and ambient air at 38°C to obtain mat growth of cyanobacterial cells. The filters were then carefully transferred to a BG-11 agar plate with 50-µg/mL kanamycin and incubated under 300 µmol·m^−2^·s^−1^ of light and 0.05% CO_2_ until the lawn growth disappeared and individual colonies appeared. A transformant colony was selected randomly and grown in 20-mL BG-11 + Kan media. Then, it was induced for 24 h with 2-g/L rhamnose and 1-mM theophylline. After induction, the culture was diluted and plated on an antibiotic plate to obtain single colonies, which were tested for deletion using tiling PCR (26), whole-genome sequencing as reported earlier (27). Primers are listed in Table S7.

### Growth characteristics

The WT and the mutant strains were grown at 38°C in 100-mL glass cultivation tubes of Multi-Cultivator photobioreactor (Photon Systems Instrument, Multi-Cultivator MC 1000, Czech Republic) containing 50-mL BG-11 media under light intensity of 1,500 (HL) µmol·m^−2^·s^−1^. The aeration provided for growth was by bubbling either 1% CO_2_ mixed air or atmospheric air through the culture. Cells were inoculated at an OD720nm of 0.025–0.1 and were cultivated to an OD of 1–1.5. The growth rate and doubling time were calculated in the exponential phase of growth. All experiments were performed in biological replicates (at least *n* = 3).

The absorption spectra of the WT and mutant strains were obtained at room temperature using a Shimadzu UV-1800 spectrophotometer. The whole-cell chlorophyll contents were calculated from the absorption spectra using the formulae obtained from Arnon et al. (39,39). The cultivated cyanobacterial cells were normalized on the basis of chlorophyll content to determine the quantum efficiency (40) of strains grown under growth condition using the FL-200 dual modulation PAM fluorometer with blue light activation as per the previous protocol (41).

### Expressing sucrose transporter in the strain with minimized genome and sucrose measurement

The sucrose over-excreting strain was constructed as reported earlier (10, 27). Briefly, the cscB gene encoding for sucrose permease [source: *E. coli* (ATCC 700927)] was integrated at the neutral site 3 of the SG55 strain and streaked on kanamycin plate for complete segregation. The sucrose productivity of the SG55-cscB strain was compared with the highest sucrose-producing strain of *Synechococcus* 2973 reported previously (10). The sucrose levels were determined following the previous protocol; briefly, cells were grown in 12-well plate for a period of 3 days along with 1-mM IPTG inducer added to the media. After 3 days, cells were harvested (OD730nm reached ~1) and centrifuged to obtain the supernatant that was used for sucrose measurement using the sucrose/d-glucose assay kit (Megazyme) (10, 27). The standard curve for sucrose and glucose was performed (27). The experiment was performed in biological (*n* = 3) and technical (*n* = 3) replicates.

## SUPPLEMENTAL MATERIAL

Supplemental material mbio.03530-23-s0001.docx Supplemental figures and tables.

ASM does not own the copyrights to Supplemental Material that may be linked to, or accessed through, an article. The authors have granted ASM a non-exclusive, world-wide license to publish the Supplemental Material files. Please contact the corresponding author directly for reuse.
