# Supplementary material for: Genome streamlining to improve performance of a fast-growing cyanobacterium Synechococcus elongatus UTEX 2973
Source: mBio. 2024 Feb 15;15(3):e03530-23. doi: 10.1128/mbio.03530-23 (PMC10936165; doi:10.1128/mbio.03530-23)
Supplement: Supplemental material — Supplemental figures and tables. [file mbio.03530-23-s0001.docx]

**Supplementary Information**

**Genome streamlining to improve performance of a fast-growing cyanobacterium *Synechococcus elongatus* UTEX 2973**

Running Title: Genome streamlining in a photoautotroph

Annesha Sengupta^a*^, Anindita Bandyopadhyay^a^*,* Debolina Sarkar^b$^, John I. Hendry^b^ , Max G. Schubert^c^, Deng Liu^a^, George M. Church^c,d^, Costas D. Maranas^b^, Himadri B. Pakrasi^a#^

*^a^Department of Biology, Washington University, St. Louis, MO, USA*

*^b^Department of Chemical Engineering, Pennsylvania State University, PA, USA*

*^c^Wyss Institute for Biologically Inspired Engineering, Harvard University, MA, USA*

*^d^Department of Genetics, Harvard Medical School, MA, USA*

^#^Corresponding author: Himadri B. Pakrasi, ﻿[pakrasi@wustl.edu](mailto:pakrasi@wustl.edu).

*Current addresses:*

*^*^Department of Chemical Engineering, University of Toronto, Canada*

*^$^International Flavors and Fragrances, 925 Page Mill Road, Palo Alto, CA 94304*

**Fig. S1:** Colony screening of SG33 strain by Tiling PCR using primers P1, P3, P4 (Primers: P1:R1_Primer 5FW and R1_Primer 6RV, P3: R1_Primer 2FW and R1_Primer 3RV, P4: R1_Primer 7FW and R1_Primer 10RV)

**
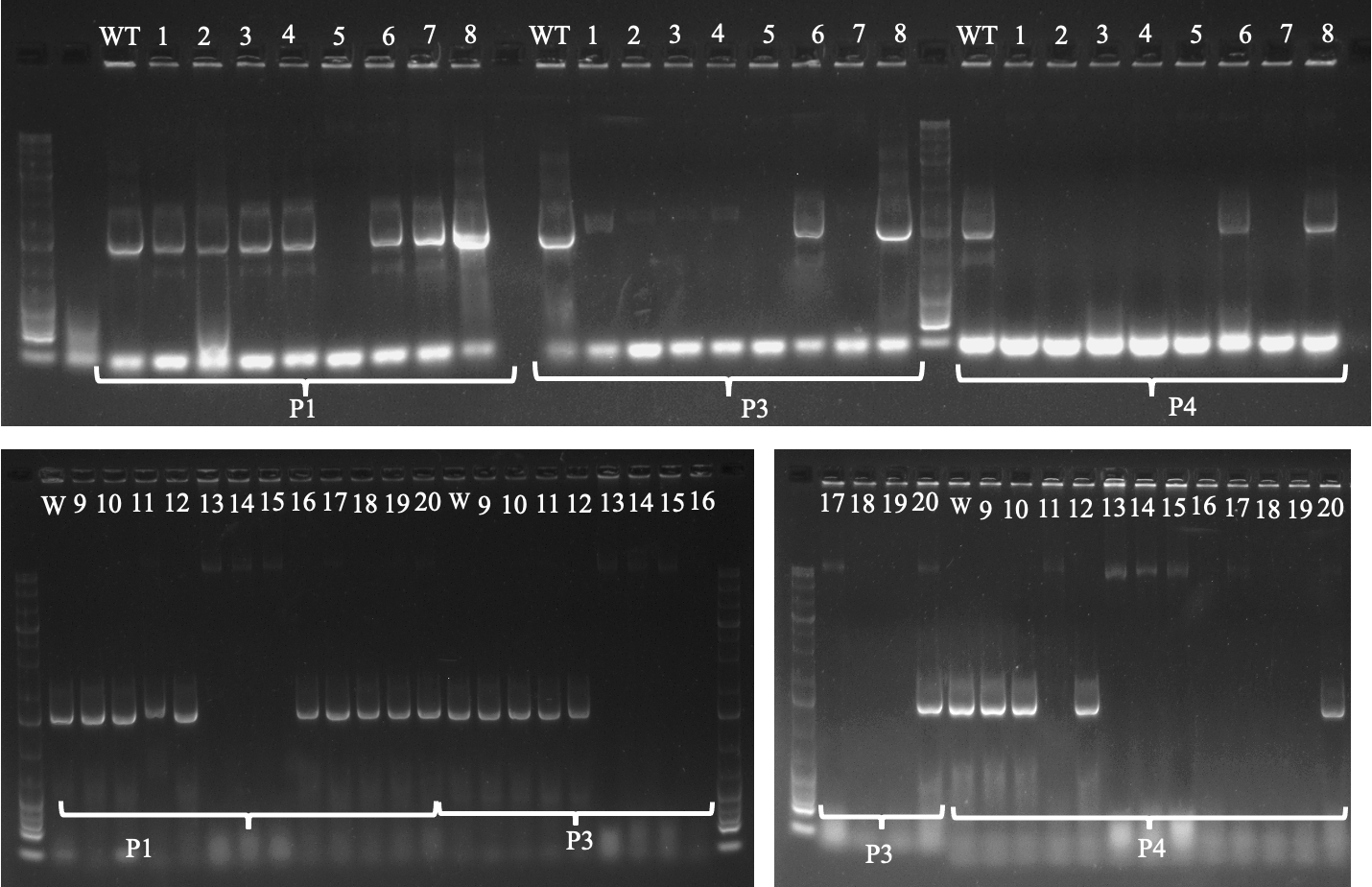
**

**Fig. S2:** Colony screening to identify positive clones for Region 3,4,5 by Tiling PCR

**
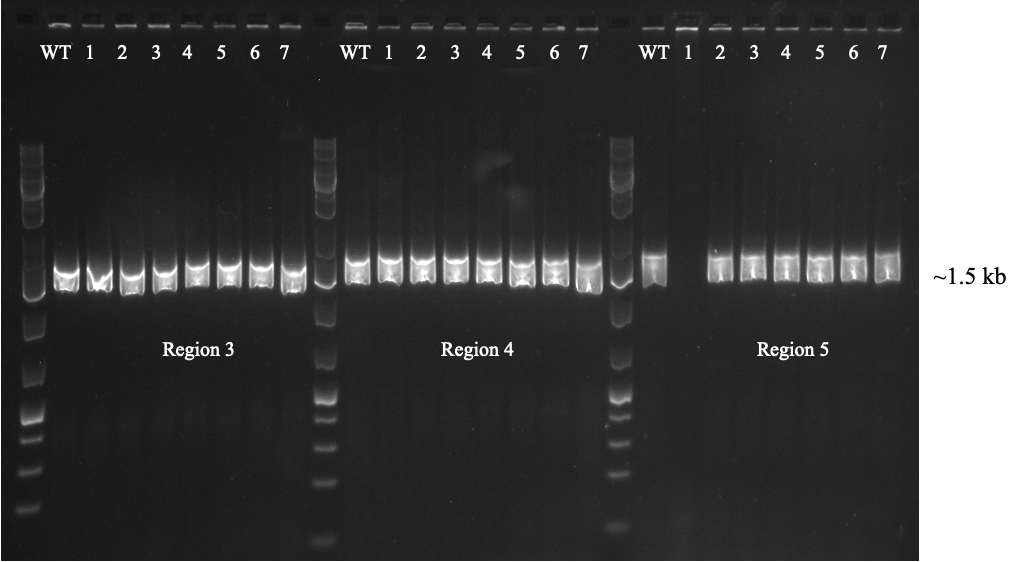
**

**Fig. S3:** Confirmation PCR for SG33 and SG20

**
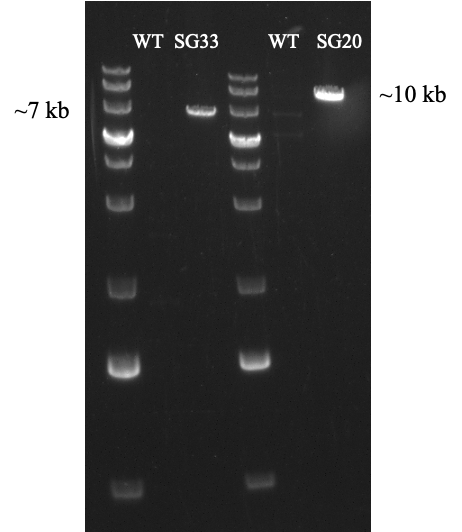
**

**Fig. S4:** Growth comparison for WT, SG33 and SG20 under high light and ambient CO_2_

**
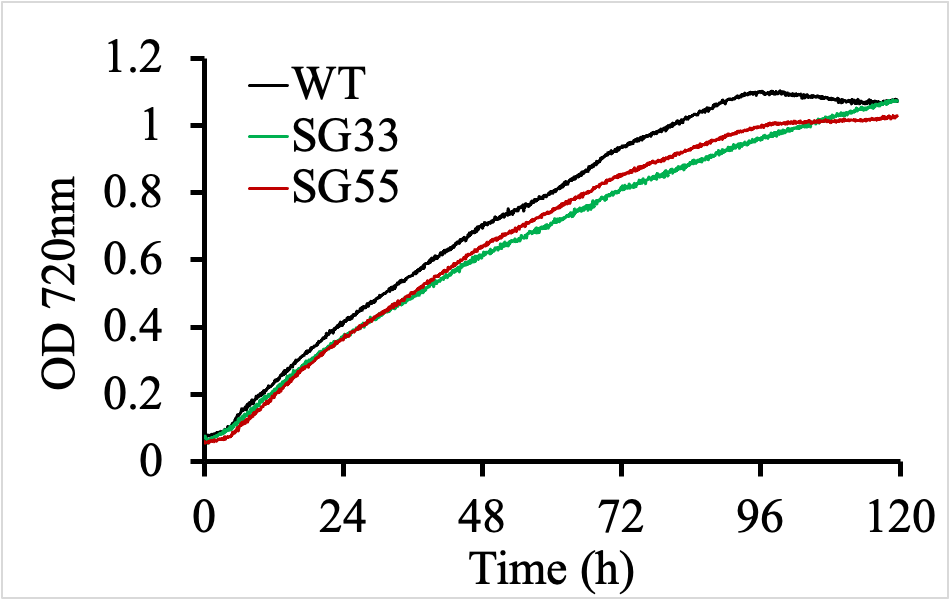
**

**Fig. S5:** Deletion of the large (pANL) and small (pANS) plasmids: CRISPR-Cas12a was employed for the task and the gel picture shows the absence of band indicating the small and large plasmids were deleted in the strains ∆pANS and ∆pANL respectively as compared to WT. Growth profile of WT and plasmid deleted strains (∆pANS and ∆pANL) under high light and high CO_2_.

**
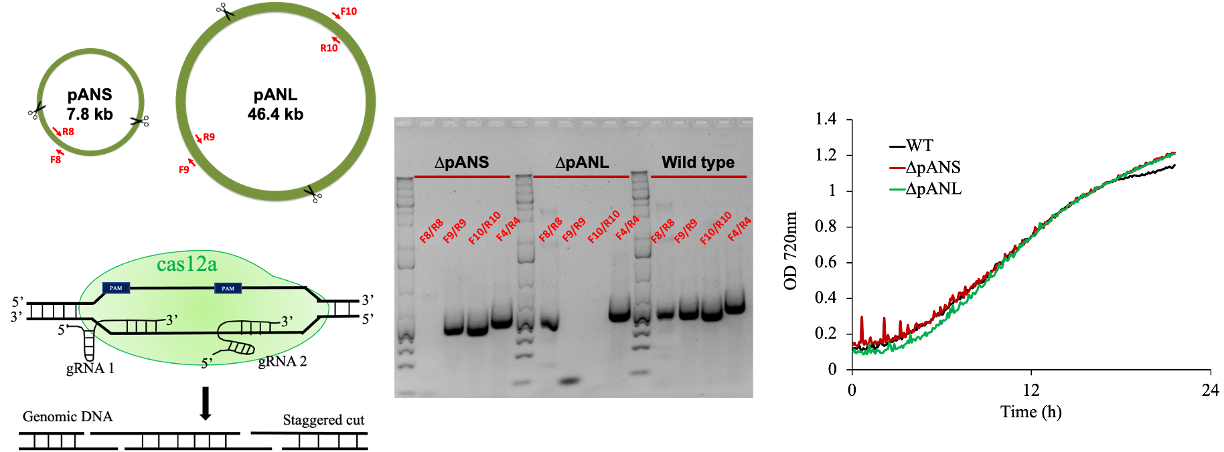
**

**Fig. S6:** The assembled plasmid map of pSL3578 showing the primer binding sites.

**
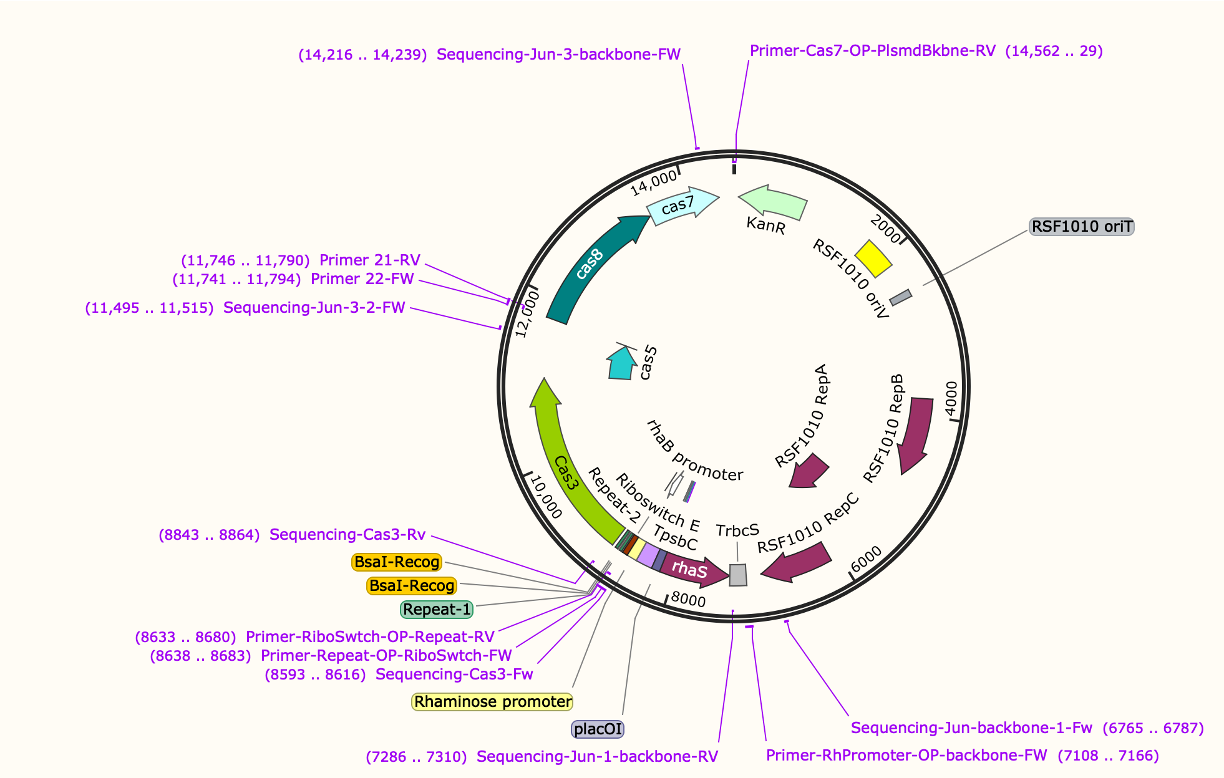
**

**Table S1:** The gene id and annotations of genes in the R1 region

| Gene | Function from Cyanobase |
| --- | --- |
| M744_12940 | hypothetical protein |
| M744_12945 | lysozyme |
| M744_12955 | hypothetical protein |
| M744_12960 | hypothetical protein |
| M744_12965 | hypothetical protein |
| M744_12970 | late control protein D |
| M744_12975 | tail protein |
| M744_12980 | hypothetical protein |
| M744_12985 | hypothetical protein |
| M744_12995 | hypothetical protein |
| M744_13000 | major tail tube protein |
| M744_13005 | hypothetical protein |
| M744_13010 | hypothetical protein |
| M744_13015 | hypothetical protein |
| M744_13020 | hypothetical protein |
| M744_13025 | hypothetical protein |
| M744_13030 | hypothetical protein |
| M744_13035 | phage tail protein |
| M744_13040 | hypothetical protein |
| M744_13045 | baseplate assembly protein W |
| M744_13050 | hypothetical protein |
| M744_13055 | baseplate assembly protein V |
| M744_13060 | hypothetical protein |
| M744_13065 | hypothetical protein |
| M744_13070 | electron transfer flavoprotein |
| M744_13075 | hypothetical protein |
| M744_13080 | hypothetical protein |
| M744_13085 | phage portal protein |
| M744_13090 | hypothetical protein |
| M744_13095 | hypothetical protein |
| M744_13100 | hypothetical protein |
| M744_13105 | hypothetical protein |
| M744_13110 | hypothetical protein |
| M744_13115 | hypothetical protein |
| M744_13120 | hypothetical protein |
| M744_13125 | helicase |
| M744_13135 | hypothetical protein |

**Table S2:** The gene id and annotations of genes in the R2 region

| Genes | Function from Cyanobase |
| --- | --- |
| M744_10800 | peptide ABC transporter substrate-binding protein |
| M744_10805 | 1-(5-phosphoribosyl)-5-amino-4-imidazole- carboxylate carboxylase |
| M744_10810 | chorismate-binding protein |
| M744_10815 | hypothetical protein |
| M744_10820 | phosphorylase |
| M744_10825 | hypothetical protein |
| M744_10830 | hypothetical protein |
| M744_10835 | hypothetical protein |
| M744_10840 | hypothetical protein |
| M744_10845 | hypothetical protein |
| M744_10850 | hypothetical protein |
| M744_10855 | transcriptional regulator |
| M744_10860 | diguanylate cyclase |
| M744_10865 | hypothetical protein |
| M744_10870 | hypothetical protein |
| M744_10875 | hypothetical protein |
| M744_10880 | hypothetical protein |
| M744_10885 | hypothetical protein |
| M744_10890 | lipoprotein |

**Table S3:** The gene id and annotations of genes in the R3 region

| Genes | Function from Cyanobase |
| --- | --- |
| M744_02780 | protein phosphatase |
| M744_02790 | glucose-1-phosphate cytidylyltransferase |
| M744_02795 | CDP-glucose 4,6-dehydratase |
| M744_02800 | hypothetical protein |
| M744_02805 | hypothetical protein |
| M744_02810 | dTDP-4-dehydrorhamnose 3,5-epimerase |
| M744_02815 | SAM-dependent methyltransferase |
| M744_02820 | glutamine--scyllo-inositol aminotransferase |
| M744_02825 | hypothetical protein |
| M744_02830 | cephalosporin hydroxylase |
| M744_02835 | hypothetical protein |
| M744_02840 | methyltransferase type 11 |
| M744_02845 | hypothetical protein |
| M744_02850 | hypothetical protein |
| M744_02855 | pili assembly chaperone |
| M744_02860 | pili assembly chaperone |
| M744_02865 | hypothetical protein |
| M744_02870 | ubiquinone biosynthesis protein |
| M744_02875 | hypothetical protein |
| M744_02880 | FAD-binding protein |
| M744_02885 | hypothetical protein |
| M744_02890 | 50S ribosomal protein L28 |
| M744_02895 | transporter |
| M744_02900 | dihydroneopterin aldolase |

**Table S4:** The gene id and annotations of genes in the R4 region

| Genes | Function from Cyanobase |
| --- | --- |
| M744_12500 | excinuclease ABC subunit A |
| M744_12505 | twitching motility protein |
| M744_12510 | hypothetical protein |
| M744_12515 | phosphotransferase |
| M744_12520 | hypothetical protein |
| M744_12525 | glutathione reductase |
| M744_12530 | FAD-dependent oxidoreductase |
| M744_12535 | hypothetical protein |
| M744_12540 | aliphatic nitrilase |
| M744_12545 | radical SAM protein |
| M744_12550 | GNAT family acetyltransferase |
| M744_12555 | selenophosphate synthetase |
| M744_12565 | ATP-dependent DNA helicase RuvB |
| M744_12570 | hypothetical protein |
| M744_12580 | hypothetical protein |
| M744_12585 | hypothetical protein |
| M744_12590 | transposase |
| M744_12595 | phosphohydrolase |
| M744_12600 | asparaginyl-tRNA synthetase |
| M744_12605 | hypothetical protein |
| M744_12610 | hypothetical protein |
| M744_12615 | cobalamin biosynthesis protein CobW |

**Table S5:** The gene id and annotations of genes in the R5 region

| Genes | Function from Cyanobase |
| --- | --- |
| M744_05410 | hypothetical protein |
| M744_05415 | sodium:proton antiporter |
| M744_05420 | hypothetical protein |
| M744_05425 | hypothetical protein |
| M744_05430 | ribonuclease H |
| M744_05435 | hypothetical protein |
| M744_05440 | Zn-dependent protease |
| M744_05445 | alkyl hydroperoxide reductase |
| M744_05450 | lipid kinase |
| M744_05455 | membrane protein |
| M744_05460 | branched-chain amino acid ABC transporter permease |
| M744_05465 | hypothetical protein |
| M744_05470 | iron deficiency-induced protein A |
| M744_05475 | Crp/Fnr family transcriptional regulator |
| M744_05480 | hypothetical protein |
| M744_05485 | hypothetical protein |
| M744_05495 | DNA polymerase III subunit beta |
| M744_05500 | Fur family transcriptional regulator |
| M744_05505 | hypothetical protein |
| M744_05510 | ATPase AAA |
| M744_05515 | hypothetical protein |
| M744_05520 | hypothetical protein |
| M744_05525 | hypothetical protein |
| M744_05530 | cell death suppressor protein Lls1 |
| M744_05535 | hypothetical protein |
| M744_05540 | hypothetical protein |
| M744_05550 | hypothetical protein |
| M744_05555 | alanine--glyoxylate aminotransferase |

**Table S6:** Doubling time of the strains used in this study under the tested conditions 1500 µmoles.m^-2^.s^-1^, 1% CO_2_ and 38˚C.

| **Strain** | **Doubling time (h)** |
| --- | --- |
| *Synechococcus* 2973 | 2.4±0.07 |
| SG33 | 1.9±0.1 |
| SG20 | 2.3±0.2 |
| SG55 | 2.2±0.12 |

**Table S7:** List of primers used in this study

| **Primer Name** | **Primer Sequence** |
| --- | --- |
| **Construction of plasmid** |  |
| Primer-Repeat-OP-RiboSwtch-FW | ctgctaaggaggtaacaacaagATGAAATTCAACTAGGTCGCGCCC |
| Primer21-RV | tgataatagtcattgagggcCGAAAGGATCATGCCTTGTCCTCTG |
| Primer22-FW | ggcggcagaggacaaggcatgatcctttCGGCCCTCAATGACTATTATCAGCGA |
| Primer-Cas7-OP-PlsmdBkbne-RV | cagagcattacgctgacttgacgggacacGGCTGACGCCGTTGGATA |
| Primer-RhPromoter-OP-backbone-FW | ataggccgctttcctggctttgcttcccacTAATTGACAATTGACAATTCCCCACTTAG |
| Primer-RiboSwtch-OP-Repeat-RV | cgcgacctagttgaatttcatCTTGTTGTTACCTCCTTAGCAGGGTGC |
| Sequencing-Cas3-Fw | gaataccggtgataccagcatcgt |
| Sequencing-Cas3-Rv | ctgcgatctgcctttaaggtgg |
| **Guide RNA** |  |
| R1-gRNA1-FW | GAAACtatcccgtttacacgatcgtcaacggtggcctgaG |
| R1-gRNA1-RV | GCGACtcaggccaccgttgacgatcgtgtaaacgggataG |
| R2-gRNA1-FW | GAAACcgtcgcaaagcagctagctaactgcagtgcctgcG |
| R2-gRNA1-RV | GCGACgcaggcactgcagttagctagctgctttgcgacgG |
| R3-gRNA1-FW | GAAACaacgtcccccaatagctttgctggatcagctcagG |
| R3-gRNA1-RV | GCGACctgagctgatccagcaaagctattgggggacgttG |
| R4-gRNA1-FW | GAAACagcagacgcgctgtgcgctggacaatatccgtcaG |
| R4-gRNA1-RV | GCGACtgacggatattgtccagcgcacagcgcgtctgctG |
| R5-gRNA1-FW | GAAACaaatcctttgccaaacagcagattgaaatgcagaG |
| R5-gRNA1-RV | GCGACtctgcatttcaatctgctgtttggcaaaggatttG |
| R5-gRNA2-FW | GAAACacaaattttgattgggacaagactgtggactcG |
| R5-gRNA2-RV | GCGACgagtccacagtcttgtcccaatcaaaatttgtG |
| R2-gRNA2-FW | GAAACaaataatgagcactcactcatctgccgcgaccggG |
| R2-gRNA2-RV | GCGACccggtcgcggcagatgagtgagtgctcattatttG |
| R3-gRNA2-FW | GAAACcaccaagagcaagctctatcaattgcaatcgcggG |
| R3-gRNA2-RV | GCGACccgcgattgcaattgatagagcttgctcttggtgG |
| R4-gRNA2-FW | GAAACcacaccactcacggggtcgaatcttaatgctgccG |
| R4-gRNA2-RV | GCGACggcagcattaagattcgaccccgtgagtggtgtgG |
| **Tiling and Confirmation PCR** |  |
| R1_TilePrimer 1FW | catagcttaggggggtgtcagg |
| R1_TilePrimer 2RV | gctgctgttcgatggcatcaa |
| R1_TilePrimer 3FW | gttgccatgcggttagatcgtc |
| R1_TilePrimer 4RV | gacgatctaaccgcatggcaac |
| R1_TilePrimer 5FW | cttgattactgcccatcatgcgg |
| R1_TilePrimer 6RV | ggccagcatcatcgaaatcaagg |
| R1_TilePrimer 7FW | gatcagcatctgccatcgcca |
| R1_TilePrimer 8RV | tggcgatggcagatgctgatc |
| R1_TilePrimer 9FW | tggactactcactcagccagcc |
| R1_TilePrimer 10RV | ggctggctgagtgagtagtcca |
| R1_TilePrimer 11FW | acccgaagcaatcagtccatg |
| R1_TilePrimer 12RV | Cttcttgtctggaagcagcagc |
| R1_TilePrimer 13FW | cgatccggtcgattgtgtccg |
| R1_TilePrimer 14RV | cggacacaatcgaccggatcg |
| R1_TilePrimer 15FW | cttcagctccttgccactgtca |
| R1_TilePrimer 16RV | tgacagtggcaaggagctgaag |
| R1_TilePrimer 17FW | gatcgtcaggtgctcgagctcg |
| R1_TilePrimer 18RV | gagctcgagcacctgacgatc |
| R1_TilePrimer 19FW | gtccagaagttttctcgccgg |
| R1_TilePrimer 20RV | ctcgtccgtgtcgaggaagg |
| R1_TilePrimer 21FW | gaaactgataggcgacctcgcg |
| R1_TilePrimer 22RV | cgctactcagtgaggccaagc |
| R1_TilePrimer 23FW | cagctctcgctgcagacacag |
| R1_TilePrimer 24RV | agcatcagtactcgcagcgtc |
| R1_TilePrimer 25FW | cgacctcagtggttgcgaag |
| R1_TilePrimer 26RV | gactctgccctggcagct |
| R1_TilePrimer 27FW | ggcgatcgcaactggcttag |
| R1_TilePrimer 28RV | ccatgacccgccttgcattaag |
| R1_TilePrimer 29FW | ctcccactctagcctcctaaatccg |
| R1_TilePrimer 30RV | acctgaacctgaagcagctaagc |
| R1_TilePrimer 31FW | cattcacaccgagcccgtcg |
| R1_TilePrimer 32RV | ccattcttcctctgcagcccaac |
| R1_TilePrimer 33FW | atctatgacgcaccaccgacg |
| R1_TilePrimer 34RV | ttggaagcgatcgatcggctttg |
| R2_TilePrimer 1FW | gcccaagtacgcctagacgttc |
| R2_TilePrimer 2RV | catccaagactcaaaggactcggtg |
| R2_TilePrimer 3FW | gactcggctgcttctgaaccg |
| R2_TilePrimer 4RV | ccctgaactagctccagacagcg |
| R2_TilePrimer 5FW | cctgctcaatcgctgcgaca |
| R2_TilePrimer 6RV | ctggcgtgaagattgttccatcg |
| R2_TilePrimer 7FW | gtggcgctcttggttcgtga |
| R2_TilePrimer 8RV | cagcttcccaaggggcaac |
| R3_TilePrimer 1FW | gatgctgaatcgccgtcacc |
| R3_TilePrimer 2RV | gcgtgctctcggctttcattc |
| R3_TilePrimer 3FW | gcgatccgtaatacggccagt |
| R3_TilePrimer 4RV | cgaatgaccgctaggctgc |
| R3_TilePrimer 5FW | ccgatacagcaactcagtttatggc |
| R3_TilePrimer 6RV | cggtccagatacgtattgctcagc |
| R3_TilePrimer 7FW | gtgattccgggacgactggc |
| R3_TilePrimer 8RV | cctcctgtctgactcatctcgc |
| R4_TilePrimer 1FW | gccctagcttgtcgagatcgag |
| R4_TilePrimer 2RV | gcgcagggcgcttttttc |
| R4_TilePrimer 3FW | ggcaatcatccaatcgatgctgg |
| R4_TilePrimer 4RV | ggcgcttggctcaagaacg |
| R4_TilePrimer 5FW | gggcgatcgcacctcaag |
| R4_TilePrimer 6RV | tgctcgagggtcagctcc |
| R4_TilePrimer 7FW | atgcccaagcgcgcttc |
| R4_TilePrimer 8RV | caggcgcgcttgaatttccc |
| R5_TilePrimer 1FW | gcgagtcattatgtacaaccgcg |
| R5_TilePrimer 2RV | ctggctagcattgacgcgat |
| R5_TilePrimer 3FW | caaatttatgcagacctcagtgccg |
| R5_TilePrimer 4RV | gccgagggtcaaacccgttt |
| R5_TilePrimer 5FW | ctaccctgaatccagcagtgagg |
| R5_TilePrimer 6RV | ccagccaagtcaccagtaaatcc |
| R5_TilePrimer 7FW | gccaaaagcttggatttgcacg |
| R5_TilePrimer 8RV | cagcagtacctacagtcaaagcc |
